# Activity budget and foraging patterns of Nubian giraffe (*Giraffa camelopardalis camelopardalis*) in Lake Nakuru National Park, Kenya

**DOI:** 10.1002/ece3.11463

**Published:** 2024-05-30

**Authors:** Consolata G. Gitau, Judith S. Mbau, Robinson K. Ngugi, Emmanuel ngumbi, Arthur B. Muneza

**Affiliations:** ^1^ Department of Land Resource Management and Agricultural Technology (LARMAT) University of Nairobi Nairobi Kenya; ^2^ Africa Fund for Endangered Wildlife (AFEW) Kenya Nairobi Kenya; ^3^ Giraffe Conservation Foundation Nairobi Kenya; ^4^ Present address: School of Animal, Rural and Environmental Sciences, Nottingham Trent University Nottingham UK

**Keywords:** activity time budget, forage selection, *Giraffa camelopardalis*, Kenya, Lake Nakuru National Park, Nubian giraffe

## Abstract

The activity budget of giraffe in various African populations has been studied extensively, revealing that it is affected by body size, foraging patterns, and sex. Foraging patterns show an animal's feeding choices in its environment and are influenced by resource availability, competition, and predation risk. The ability of giraffe to survive and reproduce is significantly impacted by the variation in activity budget and foraging across different ecosystems. Our study focused on evaluating the seasonal activity budgets and foraging patterns of Nubian giraffe in Lake Nakuru National Park, Kenya. We used the scan sampling method to record the activity budget of giraffe which included foraging, movement, resting, and drinking water. We then evaluated if activities varied with the seasons. A total of 11,280 activities were documented, with 4560 (40.4%) occurring during the dry season and 6720 (59.6%) during the wet season. Foraging accounted for 53% of the time budget during the dry season, but increased to 57% during the wet season. There was a slight drop in records of movement (22%; *n* = 995 of 4560) and resting (25%; *n* = 1145 of 4560) from the dry season to the wet season (20%; *n* = 1375 out of 6720 and 22%; *n* = 1515 of 6720). During the dry season, females (53%) foraged longer than males (47%), whereas males (44%) had longer resting periods than females (56%). Giraffe frequently fed on *Vachellia xanthophloea* (67%; *n* = 4136 of 6215 foraging records), *Maytenus senegalensis* (19%), and *Solanum incanum* (9%) over both seasons. Overall, seasons had little impact on giraffe activity time budgets and foraging patterns in Lake Nakuru National Park. A better insight into the behavioural patterns of this subspecies will allow managers to enhance the protection and conservation of the species and its habitat. Heavy foraging on *Vachellia* by giraffe at LNNP has been associated with a population decline in number, so perhaps planting more of this species in LNNP could promote a rebound in numbers.

## Graphical abstract

The activity budget of the giraffe in various African populations has been studied extensively, revealing that it is affected by body size, foraging patterns, and sex. Our study focused on evaluating the seasonal activity budgets and foraging patterns of the Nubian giraffe in Lake Nakuru National Park, Kenya using scan sampling. Overall, seasons had little impact on giraffe activity time budgets and foraging patterns in Lake Nakuru National Park.

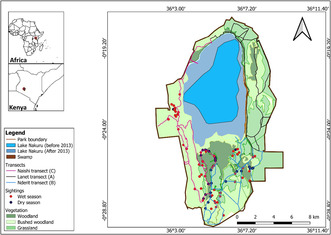

## 1 INTRODUCTION

Different animals have an activity time budget that reflects physiological traits and ecological interactions (Blake et al., 2012; Norris et al., 2010). These behavioural decisions, i.e., what activities to perform and how much time to dedicate to them can affect survival and reproductive success (Gaillard et al., 2010). An animal's decision regarding foraging, movement, resting, and drinking water might reduce the risk of predation, hence enhancing chances of survival and reproduction (Lind & Cresswell, 2005; Sansom et al., 2009). Factors such as water and food availability, temperature fluctuations, seasonal changes, growth and reproduction cycles, predator avoidance, and proximity to human settlement determine how prey species allocate their time to various behaviours (Brown et al., 2023; Owen‐Smith & Goodall, 2014; Svizzero & Tisdell, 2017; Xia et al., 2011). Animals modify their feeding behaviour by increasing the time spent foraging as the quality and quantity of browse decline during the dry season to maintain daily nutritional needs and survival (Adolfsson, 2009). Evaluating activity budgets of different species is essential for understanding how factors like forage variability with seasons affect their survival (Blanco et al., 2017; Estes et al., 2011).

Giraffe (*Giraffa* spp.) inhabit diverse habitats and consume a range of plant species such as *Vachellia, Grewia, Combretum*, and *Commiphora* spp. (Obari, 2014). Giraffe prefer *Vachellia*‐dominated savanna woodlands characterised by evenly spaced trees and open canopies that provide ample forage (Berry & Bercovitch, 2017; Ciofolo & Le Pendu, 2002; Mahenya, 2016; O'Connor et al., 2015). Berry and Bercovitch (2017) described close to 100 plant species in the diet of a population of giraffe, although only about half a dozen make up most of the forage consumed. Giraffe can thrive in regions with scarce water resources due to their water‐independent nature. However, some giraffe choose to feed near rivers to guarantee a consistent availability of food and water in all seasons (Saito & Idani, 2020; Wyatt, 1969).

Previous research has examined several aspects of giraffe behaviour, such as nocturnal behaviour (Burger et al., 2020), diurnal activity budgets (Deacon et al., 2024; Paulse et al., 2023) and the impact of seasonal changes on foraging activities (Clark et al., 2023; Pellew, 1984). Studies have shown variation in giraffe behaviour across many environments, including zoos (Fernandez et al., 2008), national parks (Lee et al., 2023; Saito & Idani, 2020), and natural habitats (Fennessy, 2009). These variations can be influenced by factors like sex differences (Bashaw, 2011; Ginnett & Demment, 1997), body size (du Toit & Yetman, 2005), anthropogenic influences (Scheijen et al., 2021), and proximity to human settlements (Bond et al., 2021). Giraffe adapt their foraging time according to food availability (Pellew, 1984), habitat utilisation patterns (Saito & Idani, 2020), and the impacts of feeding opportunities on reducing abnormal behaviour such as oral stereotypy (Enevoldsene et al., 2022). While giraffe research has been conducted throughout Africa, no equivalent research has been conducted to determine the activity pattern of Nubian giraffe (*Giraffa c. camelopardalis*) in Lake Nakuru National Park (LNNP) due to significant variations in environmental conditions.

This study seeks to better understand the ecological dynamics of LNNP, an enclosed and distinctive conservation area located on the periphery of an urban centre. Research on giraffe foraging at LNNP provides additional insights into the dietary flexibility and adaptability of giraffe, given that LNNP is on the periphery of a sprawling urban city. Urban sprawl and obstruction of giraffe dispersal patterns may limit their ability to move to suitable habitats and resources, affecting their survival and population dynamics (Bond et al., 2023). The park, like other enclosed ecosystems, has unique challenges, like habitat deterioration, competition for resources, heightened vulnerability to environmental shifts and increased risk of predation (Bond et al., 2023; Brenneman et al., 2009; Dharani et al., 2009; Gathuku et al., 2021; Muller, 2018). Giraffe need a large home range to acquire resources and restricting them to a park can affect their activity time budget and overall survival (Brenneman et al., 2009; Obari, 2014; van der Jeugd & Prins, 2000). In the Tarangire‐Manyara region of northern Tanzania, adult giraffe had an average home range size of 114.6 km^2^ for females and 157.2 km^2^ for males (Knüsel et al., 2019). The portion of LNNP not encompassed by the lake is around 134 km^2^ (Onyango, 2020). This is smaller than the typical home range size of females, although home range size is determined by resource availability (Knüsel et al., 2019). Enclosed habitats can prevent giraffe from having access to quality forage, exacerbate intraspecific competition, and/or increase the risk of predation, (Brenneman et al., 2009; Muller, 2018; Pendu & Ciofolo, 1999; van der Jeugd & Prins, 2000). Furthermore, understanding the behaviour and foraging patterns of giraffe is crucial for developing successful conservation measures due to the potential genetic isolation of giraffe populations in the region (Fennessy et al., 2013).

There are approximately 1042 Nubian giraffe in Kenya, making it the most at‐risk subspecies in the country (Muneza et al., 2024). Most of the Nubian giraffe populations in Kenya are isolated in enclosed areas (Brenneman et al., 2009; Brown et al., 2023; Dagg, 1962). Habitat degradation has reduced the Nubian giraffe habitat by 37% in sub‐Saharan Africa and 75% in Kenya (O'Connor et al., 2019). However, Bercovitch (2020) questioned these findings owing to methodological concerns. The factors affecting the Nubian giraffe activity time budget and foraging patterns in Kenya are not well known. This study aimed to evaluate how seasonal variations and sex differences could impact the activity time budget and foraging behaviour of the Nubian giraffe in LNNP, Kenya, due to the limited information available and the significance of precise data for conservation and management decisions.

## 2 STUDY AREA

LNNP covers 188 km^2^ in the Rift Valley in Nakuru County, Kenya and is situated between 00^0^ 18' S and 00^0^ 30' S latitude and 36^0^ 03′ and 36^0^ 07′ E longitude, at an elevation of around 1759 metres above sea level (KWS, 2011). The park features a central lake that covers an area of approximately 54 km^2^. The park experiences an average annual rainfall of 750 millimetres, evenly distributed between April and June and from October to December. The dry seasons occur from July to September and from January to March. The daily maximum temperature during the dry season is 28°C, while the minimum is 18°C. The average daily minimum and maximum temperatures during the wet season are 14°C and 24°C, respectively (KWS, 2011).

**FIGURE 1:**
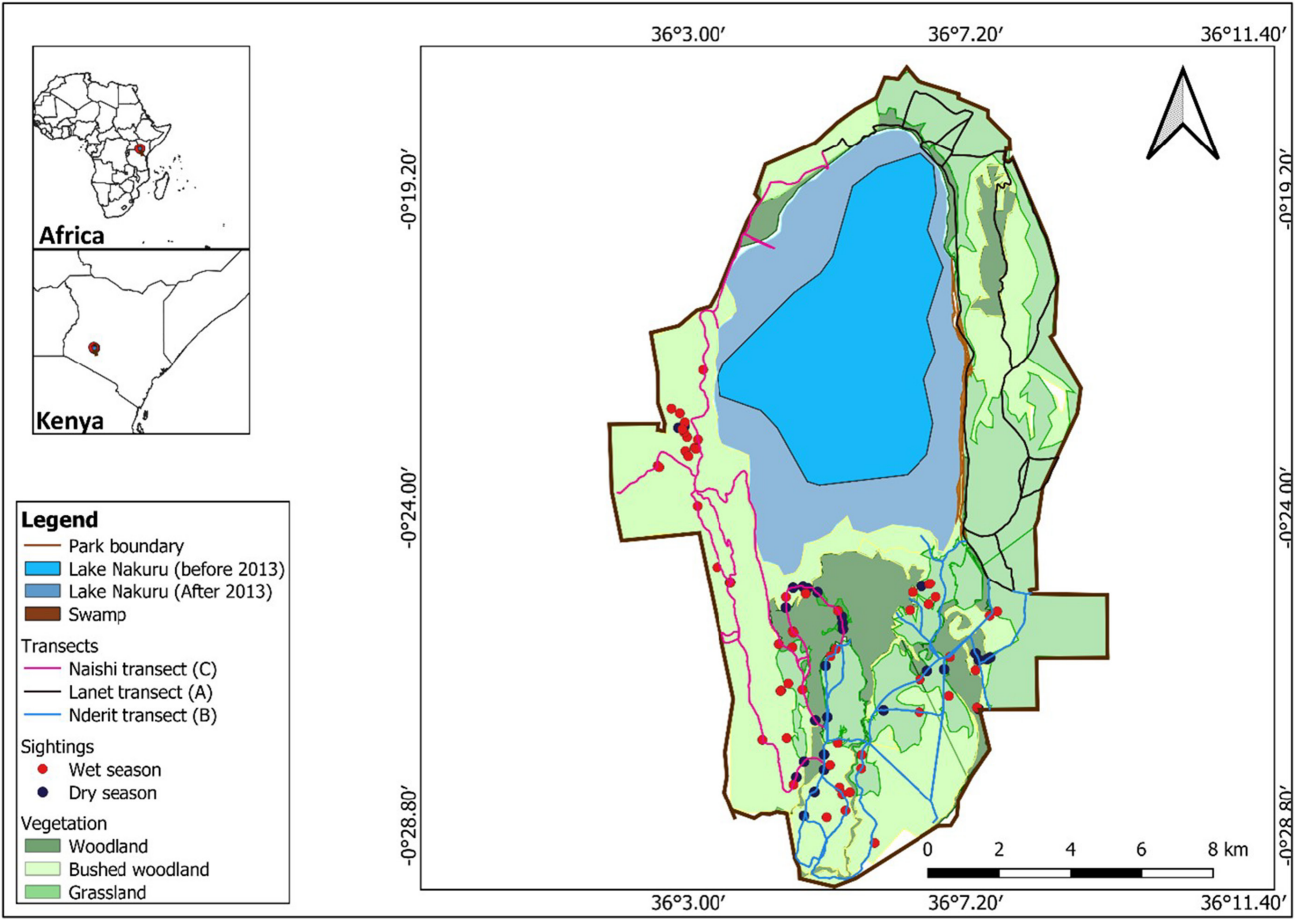
Map showing the major habitats in LNNP and the transects used for surveys of Nubian giraffe in the park.

The park comprises grasslands, woodlands, and bushed woodland as its main habitat types. *Vachellia* woodlands are widespread and associated with high water table areas (Figure 1). *Vachellia seyal*, *Vachellia hockii*, *Vachellia xanthophloea*, *Vachellia gerrardii* and *Vachellia abyssinica*, are the predominant woody species in *Vachellia* woodland. Additional species found in *Vachellia* woodland include *Achyranthes aspera*, *Solanum incanum*, and *Urtica massaica* which are short bushes as well as *Grewia similis*, *Rhus natalensis*, *Senecio lyratipartitus*, *Cassia bicapsularis*, and *Vernonia auriculifera* (Mutangah, 1994). The park's second‐largest habitat consists of bushed woodlands interspersed with *Vachellia* woodland. The primary shrub in the bushed woodlands is *Tarchonanthus camphoratus*, while the main grasses are *Cynodon dactylon* and *Sporobolus spicatus*. The main trees include *Vachellia xanthophloea* and *Vachellia gerrardii* (Mutangah, 1994). Open grasslands are located in the sedimentary plains to the north and south of the lake. The grass species present are *Chloris gayana* and *Hyparrhenia hirta*, along with *Lippia ukambensis* and *Lantana trifolia* bushes. The habitat contains scattered trees and shrubs such as *Maerua triphylla*, *Maytenus senegalensis*, *Cordia ovalis*, *Tarchonanthus camphoratus*, and *Rhus natalen*sis (Mutangah, 1994)(Data S1).

The park is home to around 400 bird species, including large colonies of lesser (*Phoeniconaias minor*) and greater (*Phoenicopterus roseus*) flamingos (McClanahan et al., 1996; Ogutu et al., 2017). The park hosts a diverse array of over 70 mammalian species, including grazers such as hippopotamus (*Hippopotamus amphibius*), waterbuck (*Kobus ellipsiprymnus*), Thompson's gazelles (*Eudorcas thomsonii*), Bohor reedbuck (*Redunca redunca*), Burchell's zebras (*Equus quagga*), warthogs (*Phacochoerus africanus*) and impala (*Aepyceros melampus*) along the lake's shoreline. *Vachellia* woodlands are inhabited by Nubian giraffe, bushbuck (*Tragelaphus sylvaticus*), black rhino (*Diceros bicornis*); grazers like cape buffalo (*Syncerus caffer*), and white rhino (*Ceratotherium simum*); mixed feeders like olive baboons (*Papio anubis*), and colobus monkey (*Colobus guereza*) and predators like leopard (*Panthera pardus*) and lion (*Panthera leo*). Bushed woodlands provide habitats for many grazers such as eland (*Taurotragus oryx*), bushbuck, impala, Chandler's mountain reedbuck (*Redunca fulvorufula*) and dik‐dik (*Madoqua kirkii*). The cliffs and escarpments are home to yellow‐spotted rock hyrax (*Heterohyrax brucei*), klipsringer (*Oreotragus oreotragus*), and Chandler's reedbuck, which are both grazers and browsers (KWS, 2002; Mutangah, 1994).

The population trend of giraffe in LNNP has been declining with a variation in recovery. The population decreased from 153 to 62 between 1998 and 2003 (Brenneman et al., 2009), then rose to 89 (Muller, 2018). The current population is estimated to be between 95 and 120 Nubian giraffe (Muneza et al., 2024). A study by Muller (2018) indicated that a high lion density of 30 lions per 100 km^2^ in the park could endanger giraffe through predation and impact their habitat selection. Unpublished data show that there were 65 lions in 2002 and 56 lions in 2010 (Bett et al., 2010; KWS, 2002). However, routine ground counts conducted between 2010 and 2017 revealed an infrequent sighting of six to sixteen individuals (Bett, unpublished data). In 2017, surveys were paired with a spatially explicit capture–recapture (SECR) method that recorded 16 lions (Elliot et al., 2020) and proposed that previous approaches may have led to double‐counting, rather than indicating a drop in the population.

### 2.1 Methods

#### 2.1.1 Giraffe activity observations

We completed pre‐survey training in November 2018 to ensure consistent and reliable recording of sightings. The park was divided into three equally sized blocks (A, B, and C) for surveys conducted along three transects: Lanet (50 km) in block A, Nderit (49 km) in block B, and Naishi (49 km) in block C. The blocks contained heterogeneous vegetation, including *Vachellia* woodland, grassland, and bushed woodland as the main types. We used roads that cut across each block as sampling lines (Figure 1).

Seven people participated in the sampling process. Three team members monitored giraffe behaviour; two took photos, and two identified plant species browsed by giraffe. We observed giraffe behaviour during the dry season from February 13 to 15 and February 20 to 22 and during the wet season from May 31 to June 2 and July 11 to 13 in 2019. Each data collection period spanned 3 days. We selected a block at random from blocks A, B, and C, and then chose the first herd we encountered from that block, sampling one block per day. Four observations were made daily, two in the morning (08:00–09:00 and 11:00–12:00) and two in the afternoon (14:00–15:00 and 17:00–18:00). Giraffe are typically most active during the early morning (07:00–09:30) and late afternoon (15:00–19:00), with less activity throughout the afternoon hours (14:00) (Leuthold & Leuthold, 1978a). Therefore, observation hours were randomly chosen to fall within these times.

We used a scan sampling method to observe Nubian giraffe daily activity, maintaining a distance of 50–100 metres from the research vehicle to avoid disturbing the animals (Altmann, 1974). We waited for 5 minutes before commencing observations to give the giraffe to acclimate to our presence. Each hour, we performed eight scans, (time taken watching a herd and writing down their activities), each lasting 5 min followed by a 20 min break after four scans. Every 5 min, three people observed and recorded the behaviour of each giraffe in the herd. The giraffe were scanned in a consistent order during the subsequent scans within each hour (left to right or right to left), and the average of the observation from the three people was calculated. We recorded the date, time, season (wet or dry), habitat type (*Vachellia* woodland, bushed woodland, or open grassland), herd size, sex, and location (GPS coordinates) at the beginning of the observation. Recorded activities included moving (running and walking), resting (lying down, standing, and socialising), foraging (biting or using the tongue to pull a leaf or twig), licking soil, and drinking. Adult giraffe were exclusively observed to enhance precision.

We recorded the plant species selection by giraffe in each herd in 5‐minute scan intervals and defined a foraging record as one plant species consumed by one giraffe during one scan. We identified the plant species selected by each giraffe based on named species, photographs, and illustrations.

### 2.2 Data analysis

We used descriptive statistics to analyse the activity budget and foraging patterns of giraffe in the study area. We calculated the percentage of time spent on each activity by averaging the frequency of each activity per hour for each season. We aggregated the foraging data for each plant species consumed each hour and expressed it as a percentage of all feeding records for that specific hour, and subsequently for each season. We calculated the percentage of occurrence of the various activities per hour and season, and the percentage of occurrence of a plant species in the foraging records for each season. We conducted a Mann–Whitney *U* test to evaluate the effect of seasonality on activity budget, seasonality and plant species in giraffe diet, and sex on activity budget. We also used the Kruskal–Wallis test to assess the effect of habitat type on the activity budget of giraffe. The dependent variables of the study were the giraffe activities (foraging, resting, moving, and drinking water) and plant species in the giraffe diet, whereas the independent variables included sex, time, season, and habitat types with the level of significance set at 0.05 (Zar, 1996). We conducted all statistical analyses using STATA version 12.0.

## 3 RESULTS

The findings of the study indicate significant variations in giraffe foraging behaviour between the dry and wet seasons. Foraging records of giraffes increased from the dry season to the wet season. Additionally, female giraffes were found to forage more frequently than male giraffe during the dry season. Habitat preference was also noted, with giraffes foraging more frequently in the *Vachellia* woodland during the dry season compared to open grassland and bushed woodland. Furthermore, giraffes foraged on eight plant species during both seasons, showing dietary diversity. Notably, there was a marked increase in the selection for *Maytenus senegalensis* in the wet season.

### 3.1 Nubian giraffe diurnal activities in the dry and wet seasons

**FIGURE 2:**
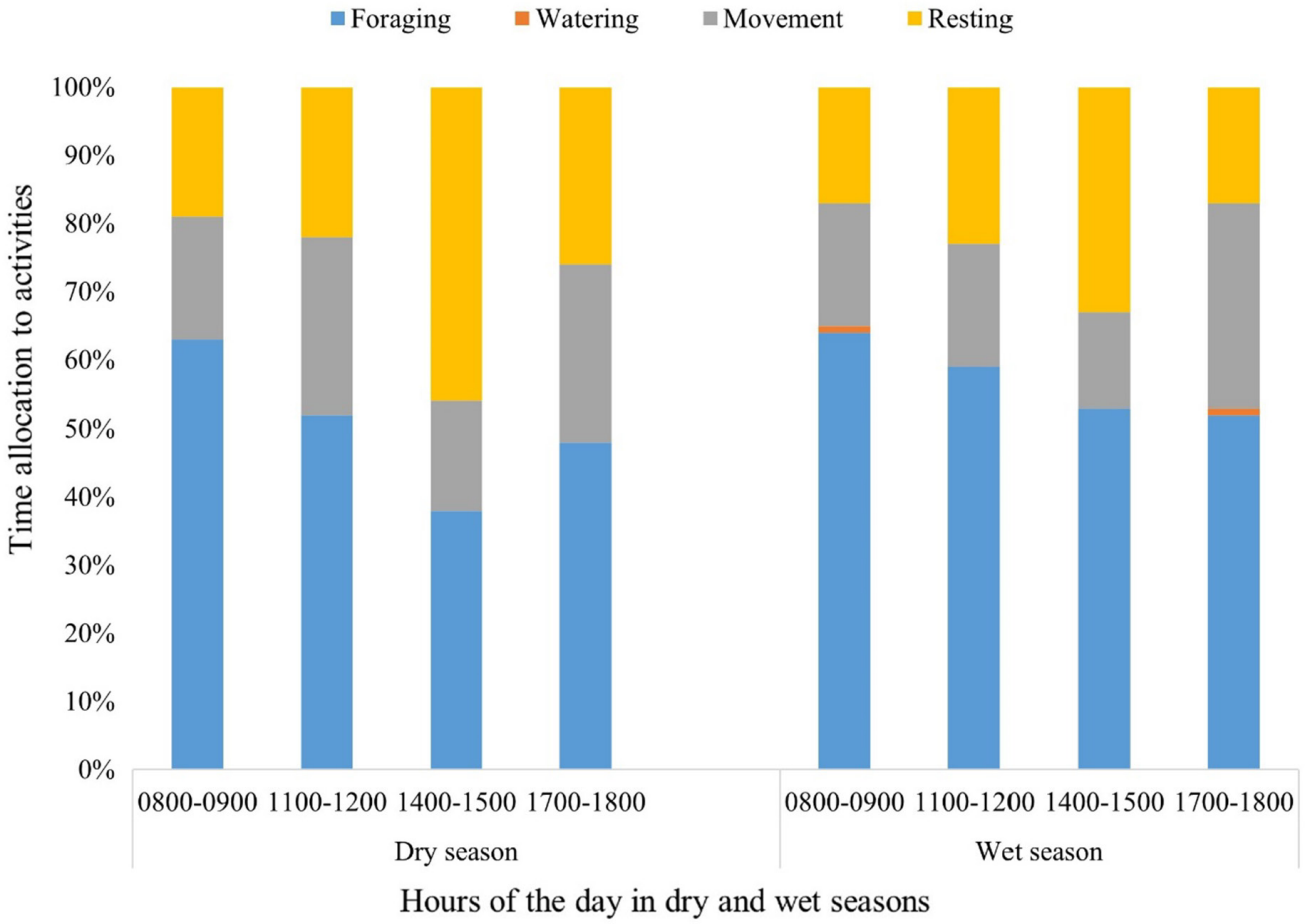
Diurnal activity time budget of the Nubian giraffe during the dry and wet seasons.

During the dry season, foraging records accounted for 53% of the activity records (Table 1). Foraging records decreased from 63% in the early morning to 52% at noon and further reduced to 38% in the afternoon. Foraging records increased to 48% in the evening (Figure 2). Movement records were lowest in the afternoon at 16%, whereas resting records were lowest in the morning at 19% (Figure 2). There were no records of giraffe drinking water during the survey period.

**TABLE 1:** Mann–Whitney *U* test results comparing the activity budget of giraffe between dry and wet seasons in LNNP.

Activities	Dry season		Wet season		*p*
	*N* = 4560		*N* = 6720		
	*n*	%	*n*	%	
Foraging	2420	53	3795	57	.091
Moving	995	22	1375	20	.307
Resting	1145	25	1515	22	.315
Watering			35	1	

Giraffe primarily engaged in foraging during the rainy season, representing around 57% of their activity (Table 1). Foraging records accounted for more than 50% of the observations while resting and moving made for 22% and 20%, respectively. From morning to evening, foraging records decreased progressively from 64% in the morning to 59% to 53% to 52% in the evening (Figure 2). Just like during the dry season, movement records were lowest in the afternoon at 14%, while the lowest resting records were made in the morning and evening. Only 1% of wet‐season mornings and evenings were spent drinking water (Figure 2).

We used the Mann–Whitney *U* test to assess the variation in giraffe activities across different seasons. We found a marginal increase in the foraging records of giraffe from the dry to the wet season accounting for 53%–57% of the total activity records (*p* = .09). Movement and resting records decreased from the dry to the wet season from 22% to 20% and 25% to 22% of the total, respectively. However, these differences were not statistically significant (*p* = .31 and *p* = .32, respectively) (Table 1). Afternoon resting records were highest in both seasons, with a greater increase observed during the dry season.

The Mann–Whitney *U* test results showed a significant difference between the frequency of giraffe foraging and resting during the dry season, based on their sex (*p* = .023 and *p* = .024, respectively) (Table 2). Female giraffe foraged more frequently (53%) compared to male giraffe (47%), while male giraffe rested more often (56%) compared to female giraffe (44%). There was no statistically significant variation in the frequency of movement of female and male giraffe throughout the dry season. No notable difference was seen in the activity budget of female and male giraffe during the wet season (Table 2).

**TABLE 2:** Mann–Whitney *U* test results comparing activity budget between female and male giraffe in LNNP.

Seasons	Activity	Sex	*n*	Mean	%	*U*	*Z*	*p*
Dry	Foraging	Female	1705	22.14	53	1046.5	−2.37	.023**
		Male	715	19.32	47			
	Moving	Female	665	8.64	49	1383	−0.26	.804
		Male	330	8.92	51			
	Resting	Female	710	9.22	44	1051	−2.34	.024**
		Male	435	11.76	56			
Wet	Foraging	Female	2845	22.58	50	2644.5	−0.01	.997
		Male	950	22.62	50			
	Moving	Female	1005	7.98	48	2338	−1.17	.261
		Male	370	8.81	52			
	Resting	Female	1155	9.17	52	2454	−0.73	.484
		Male	360	8.57	48			

**p < .05.

### 3.2 Nubian giraffe diurnal activities in different habitat types

Giraffe were predominantly found in the *Vachellia* woodland during both the dry season (60%) and wet season (63%) (Table 3). Open grassland and bushed woodland followed in order of most frequented habitats in the dry (22% and 18%) and wet seasons (21% and 16% (Table 3).

**TABLE 3:** Kruskal–Wallis test results of giraffe activity budget and habitat types in the dry and wet seasons in LNNP.

Activities	Habitat types	Dry season *N* = 4560			Wet season *N* = 6720		
		*n*	*χ* ^2^	*p*	*n*	*χ* ^2^	*p*
Foraging	Acacia woodland	1450	5.73	.06	2445	3.12	.21
	Open grasslands	495			785		
	Bushed woodlands	475			565		
Movement	Acacia woodland	575	2.56	.23	835	1.36	.51
	Open grasslands	260			305		
	Bushed woodlands	160			235		
Resting	Acacia woodland	735	1.89	.39	940	0.43	.81
	Open grasslands	245			305		
	Bushed woodlands	165			270		

During the dry season, foraging records were higher in *Vachellia* woodlands at 60% compared to the open grassland at 21% and the bushed woodland at 19% (Table 3). The Kruskal–Wallis test results showed a marginal difference between foraging and habitat types in the dry season (*p* = .06) (Table 3). During the study period, we observed that giraffe exclusively inhabited the southern half of the park throughout both the dry and wet seasons (Figure 1).

### 3.3 Plant species in giraffe diet in dry and wet seasons

We observed that giraffe foraged on *Vachellia xanthophloea*, *Maytenus senegalensis*, *Solanum incanum*, *Maerua triphylla*, *Vachellia gerrardii*, *Vachellia abyssinica*, *Rhus natalensis*, and *Grewia similis* (Table 4). In both seasons, *Vachellia xanthophloea*, *Maytenus senegalensis*, and *Solanum incanum* contributed to the bulk of the giraffe diet (Table 4).

**TABLE 4:** Mann–Whitney *U* test results of plant species in giraffe diet in the dry and wet season in LNNP.

Plant species	Dry season		Wet season		*p*
	*N* = 2420		*N* = 3795		
	*n*	%	*n*	%	
*Vachellia xanthophloea*	1743	72	2393	63	.27
*Maytenus senegalensis*	313	12	896	23	.01**
*Solanum incanum*	243	11	267	8	.48
*Maerua triphylla*	57	2	89	2	
*Vachellia gerrardii*	39	2			
*Vachellia abyssinica*			59	2	
*Rhus natalensis*			91	2	
*Grewia similis*	24	1			

**p < .05.

*Grewia similis* and *Vachellia gerrardii* were only browsed in the dry season, while *Vachellia abyssinica* and *Rhus natalensis* were browsed in the wet season (Table 4). We used the Mann–Whitney *U* test to determine whether plant species in the giraffe diet were significantly different in the dry and wet seasons. Selection for *Maytenus senegalensis* increased significantly from 12% in the dry season to 23% in the wet season (*p* = .01) (Table 4).

## 4 DISCUSSION

### 4.1 Activity budget for Nubian giraffe in Lake Nakuru National Park during the dry and wet seasons

Giraffe behaviour and activity patterns have been extensively researched, including their foraging patterns (Berry & Bercovitch, 2017; Deacon et al., 2023; Paulse et al., 2023). Multiple studies have shown a clear seasonal difference in giraffe activity budgets, with higher foraging rates during the wet seasons (Deacon, 2015; Deacon et al., 2023; Mahenya et al., 2016; Paulse et al., 2023). In the current study, we found a marginal seasonal difference in foraging between the dry and wet seasons, and no seasonal influence on resting and movement of the Nubian giraffe in LNNP. The disparity in findings prompts questions and suggests additional research to understand the reason for data divergence from previous studies. It is essential to note that individual studies may have limitations and might not always capture the full spectrum of seasonal variation in giraffe behaviour at individual and population levels. Additionally, variables like sample size, study duration, or the specific location of the study site can influence the contrasting findings. The limited number of days spent in the field was a constraint on the study. Despite these limitations, the sampling methods were chosen to capture a representative snapshot of the giraffe activity time budget within the constraints of the study's timeline and resource availability.

Giraffe showed significant sex‐dependent differences in foraging and resting activities in the dry seasons. Females foraged more frequent than males, whereas males had longer resting periods than females. Males were recorded resting more often than females, despite their bigger size, as indicated by various studies (Brand, 2007; Dagg, 1962; Paulse et al., 2023). Male individuals can optimise their energy intake by feeding in taller patches on tree species with higher leaf biomass (Brand, 2007). Other factors that could lead to the difference in foraging and resting activity between males and females include, reproductive requirement, basal metabolism, and the ability to efficiently consume low‐quality food to meet their energy demands more quickly than females (Parker, 2004; Paulse et al., 2023). We assume that male giraffe exhibited resting behaviour more frequently due to reduced foraging time, allowing for more opportunities to engage in other activities (resting and moving) (Paulse et al., 2023).

### 4.2 Nubian giraffe diurnal activity time budget in different habitat types of Lake Nakuru National Park

During the dry season, the western side of the lake, characterised by a bushed woodland habitat, was predominantly used, whereas it was largely avoided during the wet season. Giraffe primarily inhabit *Vachellia* woodlands during dry periods and spend the least amount of time in open grassland as also observed by Saito and Idani (2020). The bushed woodland habitat is characterised by fewer *Vachellia xanthophloea* trees with expansive canopies that provide more shade compared to the other habitat types (Dharani et al., 2009). Resource availability and diet quality are crucial factors influencing giraffe home ranges and habitat use, especially during periods of scarcity (Leuthold & Leuthold, 1978a, 1978b; Frost, 1996; Saito & Idani, 2020; Deacon & Smit, 2017). For instance, giraffe in Tsavo East National Park preferred habitats near rivers during the dry season and deciduous woodlands in the rainy season (Leuthold & Leuthold, 1978a, 1978b). Valeix et al. (2008) discovered that giraffe in Hwange National Park, Zimbabwe, avoided areas with little shade during hotter periods when solar radiations were at their peak. Giraffe in Katavi National Park, Tanzania, used miombo woodland during the dry season for resting due to its ample shade and capacity to accommodate larger groups (Saito & Idani, 2020). Further, other herbivores, including the giraffe, were seen to move to more open areas when wind intensity was higher for evapotranspiration‐related heat loss (Valeix et al., 2008).

Our results also showed that giraffe avoided the northern part of the park. Giraffe, just like other wild species, select habitats based on features such as the topography, and the interactions between the animals and the habitat (Godvik et al., 2009; Thurfjell et al., 2017; van Beest et al., 2016). The activity time budget and home range of wildlife, especially prey species, can be affected by the available resources, the presence of competitors, and the risk of predation, among other factors (Knüsel et al., 2019; van Beest et al., 2011). In our study area, the northern part of the park is characterised by hilly and rocky terrain, posing challenges for giraffe to inhabit the area. This is also exacerbated by the increased water levels of Lake Nakuru, which have reduced the habitat available for giraffe in the north‐western part of the park (Figure 1). The narrow strip of habitat left is also close to human settlements and the fence that was erected to keep out poachers, hence increasing the likelihood of the area being avoided.

### 4.3 Forage species in Nubian giraffe diet in dry and wet seasons

Giraffe foraged not only on the leaves of *Vachellia xanthophloea* but also on the bark, causing debarking, a behaviour that is caused by nutrient deficiency in an animal's diet (Ihwagi et al., 2012). Deacon (2015) noted that giraffe in the Kalahari region of South Africa selected plant species in the following order: *Vachellia erioloba*, *Senegalia mellifera*, *Ziziphus mucronata*, and *Boscia albitrunca* in decreasing order of selection. Milewski and Madden (2006) recorded that giraffe at Game Ranching Limited in Kenya spend the longest period feeding on *Vachellia drepanolobium*, *Vachellia seyal*, and *Balanites glabra*. Viljoen (2013) reported that 80% of the diet of a giraffe in the southwestern region of Kgalagadi Transfrontier Park, South Africa, consists of *Vachellia haematoxylon*. *Vachellia* species were seen to have the most nutritive value among 14 woody species in Zambezi National Park in Zimbabwe, with high in vitro gas production (IVGP), low acid detergent fibre (ADF), and low condensed tannin (CT) concentration, hence preferred by giraffe (Mandinyenya et al., 2019). All these observations confirm the results from this study relating to the high selection of *Vachellia* species by giraffe.

Excessive foraging on *Vachellia xanthophloea* by giraffe in LNNP was also seen from the debarking of the species, which threatens the plant species, ecosystem, and the herbivores that utilise it. Brenneman et al. (2009) stated that climatic events and the decline in flora in LNNP have led giraffe to forage more on *Vachellia* species, thus reducing the amount of quality forage. Giraffe population decline is caused by an increase in competition for the reducing browse species, as noted by Brenneman et al. (2009)). Furthermore, since 2013 the water levels of Lake Nakuru have been increasing, submerging a part of the park that has plant species fed on by giraffe. Additionally, the population of giraffe in the park has been observed to increase in recent years. Nonetheless, further research is required to determine the effects of over‐browsing on *Vachellia* species and its impacts on the giraffe population in LNNP. Giraffe numbers are increasing despite the ‘over browsing’ of *Vachellia* species and the presence of predators. Researchers need to find out whether there are enough different types of *Vachellia* species to sustain the giraffe population and whether giraffe get enough nutrients to supplement what they do not receive from *Vachellia* trees.

Giraffe foraged more on *Maytenus senegalensis* in the wet season compared to the dry season. *Maytenus senegalensis* is deciduous, thus reducing the amount of available browse for giraffes during the dry season while producing new leaves and flowers on the onset of rain (Dziba et al., 2003; Singh & Kushwaha, 2016). Further, *Maytenus senegalensis* has known medicinal values in human beings including anti‐leishmanial, antiplasmodial, antiparasitic, anti‐helminthic, and anti‐inflammatory properties (da Silva et al., 2011; Zangueu et al., 2018). We suggest that the Nubian giraffe in LNNP could be foraging on *Maytenus senegsalensis* as a form of self‐medication. However, further research is needed to understand the medicinal value of the species to the giraffe and to better understand the giraffe forage choice.

*Solanum incanum* ranked third in the giraffe diet even though it is considered a bush encroacher, toxic to livestock, a major threat to grazing, and a threat to native vegetation (Lusweti et al., 2011). The inclusion of this species in the giraffe diet could be a pointer that the woody species that giraffe feed on in the park may be lacking important minerals and nutrients required for survival (Sbhatu & Abraha, 2020). Other invasive or alien species foraged by giraffe include *Tamarix ramosissima* and *Atriplex nummularia* in the Little Karoo, South Africa (Gordon et al., 2016), and *Lantana camara* in Nairobi National Park (Obari, 2009). Foraging on invasive species can indicate the potential positive impacts these species can have on the environment and wildlife species (Chapman, 2016). Similarly, Bonanno (2016) reported that the possible benefits of invasive species are unreported and that removing them indiscriminately may not be a suitable management action.

Our findings indicate that sex significantly influences the foraging behaviour of giraffe, with females allocating more time to feeding. Our investigation also found a distinct spatial use of habitat types in LNNP among giraffe in the park. Giraffe were seen to avoid the northern section of the park throughout both dry and rainy seasons, preferring to primarily forage in the western part during the dry season. Resource availability and vegetation distribution are key elements that impact giraffe activity in the park. Lake Nakuru's rising water levels have submerged approximately 2 kilometres of the park, decreasing the giraffe's habitat. If their survival in the park is further endangered, translocation is a viable alternative to ensure their protection. The findings enhance our comprehension of giraffe behaviour and can guide conservation initiatives focused on securing appropriate habitats and resources for both male and female giraffe.

## AUTHOR CONTRIBUTIONS

**Consolata G. Gitau:** Conceptualization (equal); data curation (lead); formal analysis (lead); funding acquisition (lead); methodology (equal); project administration (equal); resources (equal); software (lead); visualization (lead); writing – original draft (lead). **Arthur B. Muneza:** Conceptualization (supporting); funding acquisition (supporting); project administration (supporting); resources (supporting); supervision (supporting); writing – review and editing (lead). **Judith S. Mbau:** Conceptualization (supporting); resources (supporting); supervision (lead); writing – review and editing (lead). **Robinson K. Ngugi:** Conceptualization (supporting); methodology (supporting); project administration (supporting); resources (supporting); supervision (supporting); validation (supporting); writing – review and editing (lead). **Emmanuel ngumbi:** Conceptualization (supporting); funding acquisition (lead); project administration (supporting); resources (supporting); validation (supporting); writing – review and editing (supporting).

## FUNDING INFORMATION

This study was funded by the African Fund for Endangered Wildlife (AFFEW) in Kenya and the Giraffe Conservation Foundation (GCF).

## CONFLICT OF INTEREST STATEMENT

The authors declare no conflict of interest.

## Supporting information


Data S1.

## Data Availability

The data that support the findings of this study are openly available in Dryad at https://datadryad.org/stash/share/HJlY2b3iGv2I_ZVvATUhyTnVjJyJBBDAnS4iPKHBNw8

## References

[ece311463-bib-0001] Adolfsson, U. G. (2009). Diurnal behaviour and utilization of shade in Masai giraffes (Giraffa camelopardalis tippelskirchi) [thesis]. Swedish University of Agricultural Sciences.

[ece311463-bib-0002] Altmann, J. (1974). Observational study of behavior: Sampling methods. Behaviour, 49(3–4), 227–266. 10.1163/156853974X00534 4597405

[ece311463-bib-0003] Bashaw, M. J. (2011). Consistency of captive giraffe behavior under two different management regimes. Zoo Biology, 30(4), 371–378. 10.1002/zoo.20338 20717898

[ece311463-bib-0004] Bercovitch, F. B. (2020). Giraffe taxonomy, geographic distribution and conservation. African Journal of Ecology, 58(2), 150–158. 10.1111/aje.12741

[ece311463-bib-0005] Berry, P. S. , & Bercovitch, F. B. (2017). Seasonal and geographical influences on the feeding ecology of giraffes in the Luangwa Valley, Z ambia: 1973–2014. African Journal of Ecology, 55(1), 80–90.

[ece311463-bib-0006] Bett, A. , Omengo, F. , & Mungai, S. (2010). Rapid survey of lions (Panthera Leo) in Lake Nakuru National Park. Kenya Wildlife Service.

[ece311463-bib-0007] Blake, J. G. , Mosquera, D. , Loiselle, B. A. , Swing, K. , Guerra, J. , & Romo, D. (2012). Temporal activity patterns of terrestial mammals in lowland rainforest of eastern Ecuador. Ecotropica, 18, 137–146.

[ece311463-bib-0008] Blanco, Y. E. D. , Spørring, K. L. , & Bitetti, M. S. D. (2017). Daily activity pattern of reintroduced giant anteaters (Myrmecophaga tridactyla): Effects of seasonality and experience. Mammalia, 81(1), 11–21. 10.1515/mammalia-2015-0088

[ece311463-bib-0009] Bonanno, G. (2016). Alien species: To remove or not to remove? That is the question. Environmental Science & Policy, 59, 67–73. 10.1016/j.envsci.2016.02.011

[ece311463-bib-0010] Bond, M. L. , König, B. , Lee, D. E. , Ozgul, A. , & Farine, D. R. (2021). Proximity to humans affects local social structure in a giraffe metapopulation. Journal of Animal Ecology, 90(1), 212–221. 10.1111/1365-2656.13247 32515083

[ece311463-bib-0011] Bond, M. L. , Lee, D. E. , & Paniw, M. (2023). Extinction risks and mitigation for a megaherbivore, the giraffe, in a human‐influenced landscape under climate change. Global Change Biology, 29(23), 6693–6712. 10.1111/gcb.16970 37819148

[ece311463-bib-0012] Brand, R. (2007). Evolutionary Ecology of Giraffes (Giraffa camelopardalis) in Etosha National Park, Namibia.

[ece311463-bib-0013] Brenneman, R. A. , Bagine, R. K. , Brown, D. M. , Ndetei, R. , & Louis, E. E. (2009). Implications of closed ecosystem conservation management: The decline of Rothschild's giraffe (Giraffa camelopardalis rothschildi) in Lake Nakuru National Park, Kenya. African Journal of Ecology, 47(4), 711–719. 10.1111/j.1365-2028.2008.01029.x

[ece311463-bib-0015] Brown, M. B. , Fennessy, J. T. , Crego, R. D. , Fleming, C. H. , Alves, J. , Brandlová, K. , Fennessy, S. , Ferguson, S. , Hauptfleisch, M. , Hejcmanova, P. , Hoffman, R. , Leimgruber, P. , Masiaine, S. , McQualter, K. , Mueller, T. , Muller, B. , Muneza, A. , O'Connor, D. , Olivier, A. J. , … Stabach, J. (2023). Ranging behaviours across ecological and anthropogenic disturbance gradients: A pan‐African perspective of giraffe (Giraffa spp.) space use. Proceedings of the Royal Society B: Biological Sciences, 290(2001), 20230912. 10.1098/rspb.2023.0912 PMC10291724 37357852

[ece311463-bib-0016] Burger, A. L. , Fennessy, J. , Fennessy, S. , & Dierkes, P. W. (2020). Nightly selection of resting sites and group behavior reveal antipredator strategies in giraffe. Ecology and Evolution, 10(6), 2917–2927. 10.1002/ece3.6106 32211165 PMC7083675

[ece311463-bib-0017] Chapman, P. M. (2016). Benefits of invasive species. Marine Pollution Bulletin, 1(107), 1–2. 10.1016/j.marpolbul.2016.04.067 27235840

[ece311463-bib-0018] Ciofolo, I. , & Le Pendu, Y. (2002). The feeding behaviour of giraffe in Niger. Mammalia, 66, 183–194. 10.1515/mamm.2002.66.2.183

[ece311463-bib-0019] Clark, R. K. , Fennessy, J. , Ferguson, S. , Fraticelli, C. , Honig, N. , Morrison, T. A. , & Brown, M. B. (2023). Seasonal dynamics impact habitat preferences and protected area use of the critically endangered Kordofan giraffe (Giraffa camelopardalis antiquorum). African journal of Wildlife Research, 53(1). 10.3957/056.053.0119

[ece311463-bib-0020] da Silva, G. , Serrano, R. , & Silva, O. (2011). Maytenus heterophylla and Maytenus senegalensis, two traditional herbal medicines. Journal of Natural Science, Biology, and Medicine, 2(1), 59–65. 10.4103/0976-9668.82320 22470236 PMC3312701

[ece311463-bib-0021] Dagg, A. I. (1962). The distribution of the giraffe in AFRICA. Mammalia, 26(4), 497–505. 10.1515/mamm-1962-0405

[ece311463-bib-0022] Deacon, F. (2015). The spatial ecology, habitat preference and diet selection of giraffe (giraffa camelopardalis giraffa) in the Kalahari region of South Africa Thesis. University of the Free State. http://scholar.ufs.ac.za/xmlui/handle/11660/1205

[ece311463-bib-0023] Deacon, F. , Smit, G. N. , & Grobbelaar, A. (2023). Resources and habitat requirements for Giraffes' (Giraffa camelopardalis) diet selection in the northwestern Kalahari, South Africa. Animals, 13(13), 2188, Article 13. 10.3390/ani13132188 37443986 PMC10339880

[ece311463-bib-0024] Deacon, F. , Smit, G. N. , & Grobbelaar, A. (2024). Diurnal activity budgets for the giraffe, Giraffa camelopardalis giraffa, in the Kalahari region of southern Africa. African Journal of Ecology, 62(2), e13252. 10.1111/aje.13252

[ece311463-bib-0025] Deacon, F. , & Smit, N. (2017). Spatial ecology and habitat use of giraffe (Giraffa camelopardalis) in South Africa. Basic and Applied Ecology, 21, 55–65. 10.1016/j.baae.2017.04.003

[ece311463-bib-0026] Dharani, N. , Kinyamario, J. I. , Wagacha, P. W. , & Rodrigues, A. J. (2009). Browsing impact of large herbivores on Acacia xanthophloea Benth in Lake Nakuru National Park, Kenya. African Journal of Ecology, 47(2), 184–191. 10.1111/j.1365-2028.2008.00954.x

[ece311463-bib-0027] du Toit, J. T. , & Yetman, C. A. (2005). Effects of body size on the diurnal activity budgets of African browsing ruminants. Oecologia, 143(2), 317–325. 10.1007/s00442-004-1789-7 15605272

[ece311463-bib-0028] Dziba, L. E. , Scogings, P. F. , Gordon, I. J. , & Raats, J. G. (2003). Effects of season and breed on browse species intake rates and diet selection by goats in the false thornveld of the eastern cape, South Africa. Small Ruminant Research, 47(1), 17–30. 10.1016/S0921-4488

[ece311463-bib-0029] Elliot, N. B. , Bett, A. , Chege, M. , Sankan, K. , de Souza, N. , Kariuki, L. , Broekhuis, F. , Omondi, P. , Ngene, S. , & Gopalaswamy, A. M. (2020). The importance of reliable monitoring methods for the management of small, isolated populations. Conservation Science and Practice, 2(7), e217. 10.1111/csp2.217

[ece311463-bib-0030] Enevoldsene, E. M. E. , Moller‐Lassesen, K. , Larsen, N. , Gert, O. E. S. , Hol Egard, R. , Jensen, S. E. , Pertoldi, C. , Jensen, T. H. , Alstrup, A. K. O. , & Johansen, K. (2022). The influence of feeding opportunities of six zoohoused Giraffa camelopardalis rothschild: The influence of feeding opportunities. Genetics & Biodiversity Journal, 6(2), 103–126. https://journals.univ‐tlemcen.dz/GABJ/index.php/GABJ/article/view/261

[ece311463-bib-0031] Estes, J. A. , Terborgh, J. , Brashares, J. S. , Power, M. E. , Berger, J. , Bond, W. J. , Carpenter, S. R. , Essington, T. E. , Holt, R. D. , Jackson, J. B. C. , Marquis, R. J. , Oksanen, L. , Oksanen, T. , Paine, R. T. , Pikitch, E. K. , Ripple, W. J. , Sandin, S. A. , Scheffer, M. , Schoener, T. W. , … Wardle, D. A. (2011). Trophic downgrading of planet earth. Science, 333(6040), 301–306. 10.1126/science.1205106 21764740

[ece311463-bib-0032] Fennessy, J. (2009). Home range and seasonal movements of Giraffa camelopardalis angolensis in the northern Namib Desert. African Journal of Ecology, 47(3), 318–327. 10.1111/j.1365-2028.2008.00963.x

[ece311463-bib-0033] Fennessy, J. , Bock, F. , Tutchings, A. , Brenneman, R. , & Janke, A. (2013). Mitochondrial DNA analyses show that Zambia's south Luangwa Valley giraffe (Giraffa camelopardalis thornicrofti) are genetically isolated. African Journal of Ecology, 51(4), 635–640. 10.1111/aje.12085

[ece311463-bib-0034] Fernandez, L. T. , Bashaw, M. J. , Sartor, R. L. , Bouwens, N. R. , & Maki, T. S. (2008). Tongue twisters: Feeding enrichment to reduce oral stereotypy in giraffe. Zoo Biology, 27(3), 200–212. 10.1002/zoo.20180 19360618

[ece311463-bib-0035] Frost, P. (1996). The Ecology of Miombo Woodlands (p. 48). Center for International Forestry Research.

[ece311463-bib-0036] Gaillard, J.‐M. , Hebblewhite, M. , Loison, A. , Fuller, M. , Powell, R. , Basille, M. , & Van Moorter, B. (2010). Habitat–performance relationships: Finding the right metric at a given spatial scale. Philosophical Transactions of the Royal Society, B: Biological Sciences, 365(1550), 2255–2265. 10.1098/rstb.2010.0085 PMC2894964 20566502

[ece311463-bib-0037] Gathuku, G. N. , Chiawo, D. O. , Warui, C. M. , Gichuki, C. M. , & Ngare, I. O. (2021). The effect of habitat type on population distribution and abundance of Rothschild's giraffe (Giraffa camelopardalis rothschildi) in Ruma National Park and Mwea National Reserve in Kenya. bioRxiv, 20210430442177. 10.1101/2021.04.30.442177

[ece311463-bib-0038] Ginnett, T. F. , & Demment, M. W. (1997). Sex differences in giraffe foraging behavior at two spatial scales. Oecologia, 110(2), 291–300. 10.1007/s004420050162 28307437

[ece311463-bib-0039] Godvik, I. M. R. , Loe, L. E. , Vik, J. O. , Veiberg, V. , Langvatn, R. , & Mysterud, A. (2009). Temporal scales, trade‐offs, and functional responses in red deer habitat selection. Ecology, 90(3), 699–710. 10.1890/08-0576.1 19341140

[ece311463-bib-0040] Gordon, C. , Eichenberger, L. , Vorster, P. , Leslie, A. , & Jacobs, S. (2016). Diet and seasonal dispersal of extralimital giraffe at Sanbona wildlife reserve, little Karoo, South Africa. Koedoe, 58, a1346. 10.4102/koedoe.v58i1.1346

[ece311463-bib-0041] Ihwagi, F. W. , Chira, R. M. , Kironchi, G. , Vollrath, F. , & Douglas‐Hamilton, I. (2012). Rainfall pattern and nutrient content influences on African elephants' debarking behaviour in Samburu and Buffalo Springs National Reserves, Kenya. African Journal of Ecology, 50(2), 152–159. 10.1111/j.1365-2028.2011.01305.x

[ece311463-bib-0042] Knüsel, M. A. , Lee, D. E. , König, B. , & Bond, M. L. (2019). Correlates of home range sizes of giraffes, Giraffa camelopardalis. Animal Behaviour, 149, 143–151. 10.1016/j.anbehav.2019.01.017

[ece311463-bib-0043] KWS, K. W. S . (2002). The status of Rothschild's giraffes (Giraffa camelopardalis rothschildi) in Lake Nakuru National Park. [Technical Report]. Kenya Wildlife Service.

[ece311463-bib-0044] KWS, K. W. S . (2011). Kenya wildlife service Lake Nakuru national park integrated management plan. Unpublished.

[ece311463-bib-0045] Lee, D. E. , Lohay, G. G. , Madeli, J. , Cavener, D. R. , & Bond, M. L. (2023). Masai giraffe population change over 40 years in Arusha national park. African Journal of Ecology, 61(2), 345–353. 10.1111/aje.13115

[ece311463-bib-0046] Leuthold, B. M. , & Leuthold, W. (1978a). Daytime activity patterns of gerenuk and giraffe in Tsavo National Park, Kenya. African Journal of Ecology, 16(4), 231–243. 10.1111/j.1365-2028.1978.tb00444.x

[ece311463-bib-0047] Leuthold, B. M. , & Leuthold, W. (1978b). Ecology of the giraffe in Tsavo east National Park, Kenya. African Journal of Ecology, 16(1), 1–20. 10.1111/j.1365-2028.1978.tb00419.x

[ece311463-bib-0048] Lind, J. , & Cresswell, W. (2005). Determining the fitness consequences of antipredation behavior. Behavioral Ecology, 16(5), 945–956. 10.1093/beheco/ari075

[ece311463-bib-0049] Lusweti, A. , Wabuyele, E. , Ssegawa, P. , & Mauremootoo, J. R. (2011). Invasive plants of East Africa (Kenya, Uganda and Tanzania) (3.5 Key and Key Sheets Lucid v). Makerere University. keys.lucidcentral.org/keys/v3/EAFRINET

[ece311463-bib-0050] Mahenya, O. (2016). Browsing by giraffe in heterogenous savanna [dissertation]. Inland Norway University of Applied Sciences.

[ece311463-bib-0051] Mahenya, O. , Mathisen, K. M. , Andreassen, H. P. , & Skarpe, C. (2016). Hierarchical foraging by giraffe in a heterogeneous savannah, Tanzania. African Journal of Ecology, 54(2), 136–145. 10.1111/aje.12270

[ece311463-bib-0052] Mandinyenya, B. , Monks, N. , Mundy, P. J. , Sebata, A. , & Chirima, A. (2019). Habitat use by giraffe and greater kudu in the Zambezi National Park, Zimbabwe. African Journal of Ecology, 57(2), 286–289.

[ece311463-bib-0053] McClanahan, Y. , Young, A. P. , at the L. C. C. T. P , Young, T. P. , & McClanahan, S. C. Z. T. (1996). East African ecosystems and their conservation. Oxford University Press.

[ece311463-bib-0054] Milewski, A. V. , & Madden, D. (2006). Interactions between large African browsers and thorny acacia on a wildlife ranch in Kenya. African Journal of Ecology, 44(4), 515–522. 10.1111/j.1365-2028.2006.00665.x

[ece311463-bib-0055] Muller, Z. (2018). Population structure of giraffes is affected by management in the great Rift Valley, Kenya. PLoS One, 13(1), e0189678. 10.1371/journal.pone.0189678 29298338 PMC5751992

[ece311463-bib-0056] Muneza, A. B. , Kavutha, J. S. , Muruana, M. W. , Ikime, T. , Kariuki, L. , Lekolool, I. , Fennessy, S. , Bett, A. , Kipchumba, A. K. , Ngumbi, E. , & Fennessy, J. (2024). Updated review of the conservation status of Nubian giraffe (Giraffa camelopardalis camelopardalis) in Kenya. Biodiversity and Conservation, 33(4), 1269–1284. 10.1007/s10531-024-02824-x

[ece311463-bib-0057] Mutangah, J. G. (1994). The vegetation of Lake Nakuru National Park, Kenya: A synopsis of the vegetation types with annotated species list. Journal of East African Natural History, 83(1), 71–96. 10.2982/0012-8317(1994)83[71:TVOLNN]2.0.CO;2

[ece311463-bib-0058] Norris, D. , Michalski, F. , & Peres, C. A. (2010). Habitat patch size modulates terrestrial mammal activity patterns in Amazonian forest fragments. Journal of Mammalogy, 91(3), 551–560. 10.1644/09-MAMM-A-199.1

[ece311463-bib-0059] Obari, T. (2014). Population ecology of Maasai giraffe (giraffa camelopardalis tippelskirchi) in relation to climate variability in southern Kenya [Dissertation]. University of Nairobi.

[ece311463-bib-0060] Obari, T. O. (2009). Factors affecting habitat use by Masai giraffe (Giraffa camelopardalis tippelskirchi L) in Athi‐Kapiti plains ecosystems, Kenya, [Thesis]. University of Nairobi.

[ece311463-bib-0061] O'Connor, D. , Stacy‐Dawes, J. , Muneza, A. , Fennessy, J. , Gobush, K. , Chase, M. J. , Brown, M. B. , Bracis, C. , Elkan, P. , Zaberirou, A. R. M. , Rabeil, T. , Rubenstein, D. , Becker, M. S. , Phillips, S. , Stabach, J. A. , Leimgruber, P. , Glikman, J. A. , Ruppert, K. , Masiaine, S. , & Mueller, T. (2019). Updated geographic range maps for giraffe, Giraffa spp., throughout sub‐Saharan Africa, and implications of changing distributions for conservation. Mammal Review, 49(4), 285–299. 10.1111/mam.12165

[ece311463-bib-0062] O'Connor, D. A. , Butt, B. , & Foufopoulos, J. B. (2015). Foraging ecologies of giraffe (Giraffa camelopardalis reticulata) and camels (Camelus dromedarius) in northern Kenya: Effects of habitat structure and possibilities for competition? African Journal of Ecology, 53(2), 183–193.

[ece311463-bib-0063] Ogutu, J. O. , Kuloba, B. , Piepho, H.‐P. , & Kanga, E. (2017). Wildlife population dynamics in human‐dominated landscapes under community‐based conservation: The example of Nakuru wildlife conservancy, Kenya. PLoS One, 12(1), e0169730. 10.1371/journal.pone.0169730 28103269 PMC5245813

[ece311463-bib-0064] Onyango, H. P. (2020). Mapping the flooding of Lake Nakuru National Park and its Effects on Resident Wildlife Thesis. University of Nairobi. http://erepository.uonbi.ac.ke/handle/11295/152952

[ece311463-bib-0065] Owen‐Smith, N. , & Goodall, V. (2014). Coping with savanna seasonality: Comparative daily activity patterns of African ungulates as revealed by GPS telemetry. Journal of Zoology, 293, 181–191. 10.1111/jzo.12132

[ece311463-bib-0066] Parker, M. P. (2004). The feeding biology and potential impact of introduced giraffe (giraffa camelopardalis) in the eastern cape province, South Africa—GCF resource library. https://library.giraffeconservation.org/download/the‐feeding‐biology‐and‐potential‐impact‐of‐introduced‐giraffe‐giraffa‐camelopardalis‐in‐the‐eastern‐cape‐province‐south‐africa/

[ece311463-bib-0067] Paulse, J. , Couldridge, V. , Cupido, C. , & Deacon, F. (2023). The diurnal activity budgets of extralimital giraffe (Giraffa camelopardalis giraffa) in the Western Cape Province, South Africa. African Journal of Ecology, 61(3), 746–751. 10.1111/aje.13135

[ece311463-bib-0068] Pellew, R. A. (1984). Food consumption and energy budgets of the giraffe. Journal of Applied Ecology, 21(1), 141–159. JSTOR. 10.2307/2403043

[ece311463-bib-0069] Pendu, Y. L. , & Ciofolo, I. (1999). Seasonal movements of giraffes in Niger. Journal of Tropical Ecology, 15(3), 341–353. 10.1017/S0266467499000863

[ece311463-bib-0070] Saito, M. , & Idani, G. (2020). Giraffe diurnal recumbent behavior and habitat utilization in Katavi National Park, Tanzania. Journal of Zoology, 312(3), 183–192. 10.1111/jzo.12825

[ece311463-bib-0071] Sansom, A. , Lind, J. , & Cresswell, W. (2009). Individual behavior and survival: The roles of predator avoidance, foraging success, and vigilance. Behavioral Ecology, 20(6), 1168–1174. 10.1093/beheco/arp110

[ece311463-bib-0072] Sbhatu, D. B. , & Abraha, H. B. (2020). Preliminary antimicrobial profile of *Solanum incanum* L.: A common medicinal plant. Evidence‐based Complementary and Alternative Medicine, 2020, e3647065. 10.1155/2020/3647065 PMC6996673 32063983

[ece311463-bib-0073] Scheijen, C. P. J. , van der Merwe, S. , Ganswindt, A. , & Deacon, F. (2021). Anthropogenic influences on distance traveled and vigilance behavior and stress‐related endocrine correlates in free‐roaming giraffes. Animals, 11(5) Article 5, 1239. 10.3390/ani11051239 33923117 PMC8145588

[ece311463-bib-0074] Singh, K. P. , & Kushwaha, C. P. (2016). Deciduousness in tropical trees and its potential as indicator of climate change: A review. Ecological Indicators, 69, 699–706. 10.1016/j.ecolind.2016.04.011

[ece311463-bib-0075] Svizzero, S. , & Tisdell, C. (2017). Were desert kites used exclusively as driven hunting structures? Unresolved issues and alternative interpretations of the evidence‐socio‐economic and biological considerations (a draft) (13278231). AgEcon Search.

[ece311463-bib-0076] Thurfjell, H. , Ciuti, S. , & Boyce, M. S. (2017). Learning from the mistakes of others: How female elk (Cervus elaphus) adjust behaviour with age to avoid hunters. PLoS One, 12(6), e0178082. 28614406 10.1371/journal.pone.0178082 PMC5470680

[ece311463-bib-0077] Valeix, M. , Fritz, H. , Matsika, R. , Matsvimbo, F. , & Madzikanda, H. (2008). The role of water abundance, thermoregulation, perceived predation risk and interference competition in water access by African herbivores. African Journal of Ecology, 46(3), 402–410. 10.1111/j.1365-2028.2007.00874.x

[ece311463-bib-0078] van Beest, F. M. , McLoughlin, P. D. , Mysterud, A. , & Brook, R. K. (2016). Functional responses in habitat selection are density dependent in a large herbivore. Ecography, 39(6), 515–523. 10.1111/ecog.01339

[ece311463-bib-0079] van Beest, F. M. , Rivrud, I. M. , Loe, L. E. , Milner, J. M. , & Mysterud, A. (2011). What determines variation in home range size across spatiotemporal scales in a large browsing herbivore? Journal of Animal Ecology, 80(4), 771–785. 10.1111/j.13e65-2656.2011.01829.x 21388373

[ece311463-bib-0080] van der Jeugd, H. P. , & Prins, H. H. T. (2000). Movements and group structure of giraffe (Giraffa camelopardalis) in Lake Manyara National Park, Tanzania. Journal of Zoology, 251(1), 15–21. 10.1017/S0952836900005033

[ece311463-bib-0081] Viljoen, S. (2013). Habitat use and diet preference of extralimital giraffes in the Kgalagadi Transfrontier Park. https://open.uct.ac.za/bitstream/handle/11427/14013/thesis_sci_2013_viljoen_giraffe_diet.pdf?sequence=1

[ece311463-bib-0082] Wyatt, J. R. (1969). The feeding ecology of giraffe (giraffa camelopardalis linnaeus) in nairobi national park, and the effect of browsing on their main food plants. Thesis. University of Nairobi. http://erepository.uonbi.ac.ke/handle/11295/25719

[ece311463-bib-0083] Xia, C. , Yang, W. , Blank, D. , Xu, W. , Qiao, J. , & Liu, W. (2011). Diurnal time budget of goitred gazelles (Gazella subgutturosa Güldenstaedt, 1780) in Xinjiang, China. Mammalia, 75(3), 235–242. 10.1515/mamm.2011.020

[ece311463-bib-0084] Zangueu, C. B. , Olounlade, A. P. , Ossokomack, M. , Djouatsa, Y. N. N. , Alowanou, G. G. , Azebaze, A. G. B. , Llorent‐Martínez, E. J. , de Córdova, M. L. F. , Dongmo, A. B. , & Hounzangbe‐Adote, M. S. (2018). In vitro effects of aqueous extract from Maytenus senegalensis (lam.) Exell stem bark on egg hatching, larval migration and adult worms of Haemonchus contortus. BMC Veterinary Research, 14, 147. 10.1186/s12917-018-1475-3 29716590 PMC5930434

[ece311463-bib-0085] Zar, J. H. (1996). Biostatistical analysis, 3 edPrentice‐Hall ((3rd ed)). Pearson Education India.

