# Supplementary material for: Activity budget and foraging patterns of Nubian giraffe (Giraffa camelopardalis camelopardalis) in Lake Nakuru National Park, Kenya
Source: Ecol Evol. 2024 May 30;14(6):e11463. doi: 10.1002/ece3.11463 (PMC11139672; doi:10.1002/ece3.11463)
Supplement: Supplementary file 1 — Data S1. [file ECE3-14-e11463-s001.docx]

**SUPPLEMENTAL METHOD SECTION**

**Plant species in the study area**

Woody species like *Vachellia seyal* ((Del.) P.J.H.Hurter, *Vachellia hockii* (De Wild.) Seigler and Ebinger), *Vachellia xanthophloea* (Benth.) P.J.H. Hurter, *Vachellia gerrardii* (Benth.) P.J.H. Hurter, and *Vachellia abyssinica* (Hochst. ex. Benth.) Kyal. and Boatwr, dominate *Vachellia* woodland. *Achyranthes aspera* (L.), *Solanum incanum* (Ruiz and Pav.), and *Urtica massaica* (Mildbr.) are short bushes; *Grewia similis* (K.Schum.), *Rhus natalensis* (Bernh. ex Krauss), *Senecio lyratipartitus* (Forssk.), *Cassia bicapsularis* (L.), and *Vernonia auriculifera* (Hiern) (Mutangah, 1994). Bushed woodlands, which alternate with *VachelliaVachellia* woodland, are the park’s second-largest ecosystem. *Tarchonanthus camphoratus* (Houtt. ex DC., Prod.) is the main shrub, *Cynodon dactylon* (Caro and Sánchez) Romero Zarco, Lagascalia, and *Sporobolus spicatus* (Vahl) Kunth are the main grasses, *Vachellia xanthophloea* and *Vachellia gerardii* are the main trees (Mutangah, 1994). Open grasslands are found in the sedimentary plains north and south of the lake. The grasslands are *Chloris gayana* (Kunth.) and *Hyparrhenia hirta* (Troupin, Fl. Garamba), with *Lippia ukambensis* ((Vatke) Verdc) and *Lantana trifolia* bushes (L.). The habitat has scattered trees and shrubs, including *Vachellia xanthophloea*, *Vachellia gerrardii*, *Vachellia hockii*, *Maerua triphylla* (A.Rich.), *Maytenus senegalensis*, *Cordia ovalis* (R.Br., Salt., Voy. Abyss. Append., *Tarchonanthus camphoratus*, *Rhus natalen*sis (Mutangah, 1994).
